# Complete mitochondrial genome sequence of *Anax parthenope* (Odonata: Anisoptera: Aeshnidae) and phylogenetic analysis

**DOI:** 10.1080/23802359.2020.1848479

**Published:** 2021-01-12

**Authors:** Ying Wang, Yimin Du, Xiang Song, Aijun Huang

**Affiliations:** a School of Life Sciences, Gannan Normal University, Ganzhou, China; b National Navel Orange Engineering and Technology Research Center, Ganzhou, China

**Keywords:** Odonata, Aeshnidae, mitochondrial genome, *Anax parthenope*, phylogenetic analysis

## Abstract

*Anax parthenope* (Odonata: Aeshnidae) is a big dragonfly which can be seen patrolling around ponds, lakes and other still water. In this study, we sequenced and analyzed the complete mitochondrial genome (mitogenome) of *A. parthenope*. This mitogenome was 15,366 bp long and encoded 13 protein-coding genes (PCGs), 22 transfer RNA genes (tRNAs) and two ribosomal RNA unit genes (rRNAs). The nucleotide composition of the mitogenome was biased toward A and T, with 74.8% of A + T content (A 40.1%, T 34.7%, C 14.0%, G 11.2%). Gene order was conserved and identical to most other previously sequenced Aeshnidae dragonflies. Most PCGs of *A. parthenope* have the conventional start codons ATN (six ATG, three ATT, and two ATC), with the exception of *cox1* and *nad1* (TTG). Except for three genes (*cox1*, *cox2*, and *nad5*) end with the incomplete stop codon T––, all other PCGs terminated with the stop codon TAA. Phylogenetic analysis showed that *A. parthenope* is sister to *Anax imperator* with high support value. All 15 Anisoptera species constituted a major clade with well support, and Aeshnidae had a close relationship with Gomphidae and Libellulidae.

Aeshnidae (Odonata: Anisoptera) is a diverse group with a worldwide distribution. This family comprises 50 genera with approximately 384 species, and all are excellent vagile fliers with confluent compound eyes and a prominent ovipositor (Von Ellenrieder 2002). *Anax parthenope* (Selys 1839), a dragonfly of the family Aeshnidae, is found in south Europe, north Africa, and Asia. This species has a blue saddle which can be seen in flight at the abdomen and the eyes are green. It is most commonly seen from June to September but can still be around in November. After mating, eggs of *A. parthenope* are inserted into plants or in mud and hatch out in 2 months while larval development takes 2 years.

Specimens of *A. parthenope* were collected from Nanyang City, Henan Province, China (33°10′N, 111°32′E, July 2019) and were stored in Entomological Museum of Gannan Normal University (Accession number GNU-EAP05). Total genomic DNA was extracted from tissues using the DNeasy DNA Extraction kit (Qiagen, Hilden, Germany). A pair-end sequence library was constructed and sequenced using Illumina HiSeq 2500 platform (Illumina, San Diego, CA), with 150 bp pair-end sequencing method. A total of 24.8 million reads were generated and had been deposited in the NCBI Sequence Read Archive (SRA) with accession number SRR12805571. Raw reads were assembled using MITObim v 1.7 (Hahn et al. 2013). By comparison with the homologous sequences of other Aeshnidae species from GenBank, the mitogenome of *A. parthenope* was annotated using software GENEIOUS R11 (Biomatters Ltd., Auckland, New Zealand).

The complete mitogenome of *A. parthenope* is 15,366 bp in length (GenBank accession no. MT371045), and containing the typical set of 13 protein-coding, 2 rRNA, and 22 tRNA genes, and one non-coding AT-rich region. The nucleotide composition of the mitogenome was biased toward A and T, with 74.8% of A + T content (A 40.1%, T 34.7%, C 14.0%, G 11.2%). Most PCGs of *A. parthenope* have the conventional start codons ATN (six ATG, three ATT, and two ATC), with the exception of *cox1* and *nad1* (TTG). Except for three genes (*cox1*, *cox2*, and *nad5*) end with the incomplete stop codon T−, all other PCGs terminated with the stop codon TAA. The 22 tRNA genes vary from 65 bp (*trnC* and *trnG*) to 72 bp (*trnK*). Two rRNA genes (*rrnL* and *rrnS*) locate at *trnL1*/*trnV* and *trnV*/control region, respectively. The lengths of *rrnL* and *rrnS* in *A. parthenope* are 1,299 and 773 bp, with the AT contents of 76.9% and 74.4%, respectively. Gene order was conserved and identical to most other previously sequenced Aeshnidae dragonflies (Yong et al. 2016; Kim et al. 2018; Guan et al. 2019; Lan et al. 2019; Wang et al. 2019).

Phylogenetic analysis was performed based on the nucleotide sequences of 13 PCGs from 23 Odonata species. The phylogenetic tree was constructed through raxmlGUI 1.5 (Silvestro and Michalak 2012). Results showed that the new sequenced species *A. parthenope* is sister to *Anax imperator* with a high support value (Figure 1). All 15 Anisoptera species constituted a major clade with good support, indicating the monophyly of Anisoptera could be confirmed by the PCGs dataset in this analysis. Within Anisoptera, Aeshnidae had a close relationship with Gomphidae and Libellulidae, similar results were found in the previous work (Kim et al. 2018). In conclusion, the mitogenome of *A. parthenope* sequenced in this study and provide essential and important DNA molecular data for further phylogenetic and evolutionary analysis of Aeshnidae.

**Figure 1.**
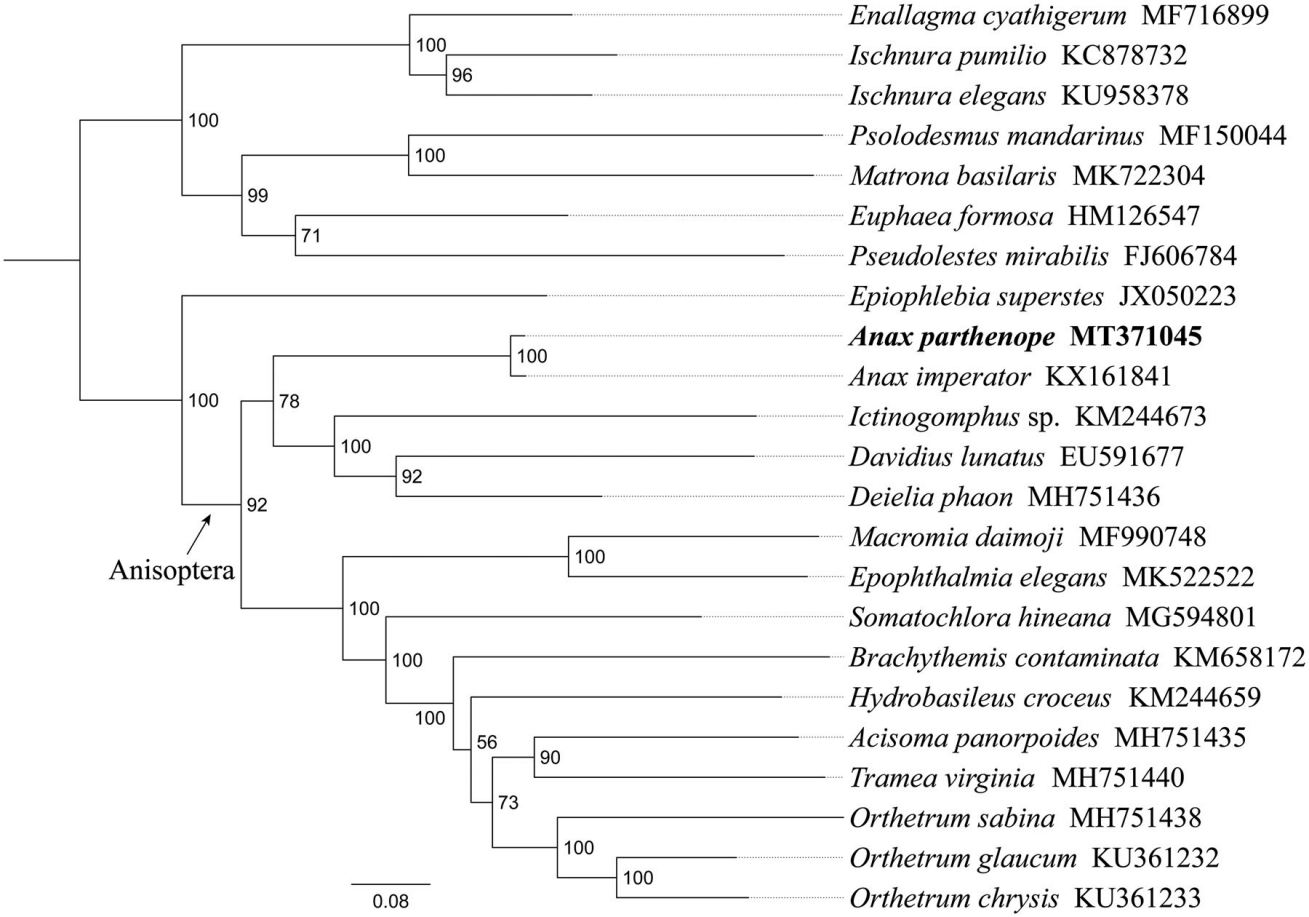
Phylogenetic relationships based on the 13 mitochondrial protein-coding genes sequences inferred from RaxML. Numbers on branches are bootstrap support values (BS).

## Data Availability

Mitogenome data supporting this study are openly available in GenBank at: https://www.ncbi.nlm.nih.gov/nuccore/MT371045. Associated BioProject, SRA, and BioSample accession numbers are https://www.ncbi.nlm.nih.gov/bioproject/PRJNA668436, https://www.ncbi.nlm.nih.gov/sra/SRR12805571, and https://www.ncbi.nlm.nih.gov/biosample/SAMN16409369, respectively.
